# Amino acid fortified diets for weanling pigs replacing fish meal and whey protein concentrate: Effects on growth, immune status, and gut health

**DOI:** 10.1186/2049-1891-5-57

**Published:** 2014-12-22

**Authors:** Yan Zhao, Alexandra C Weaver, Vivek Fellner, Robert L Payne, Sung Woo Kim

**Affiliations:** Department of Animal Science, North Carolina State University, Raleigh, NC 27695 USA; Evonik Corp, Kennesaw, GA 30144 USA

**Keywords:** Amino acid, Fish meal, Growth performance, Pig, Whey protein concentrate

## Abstract

**Background:**

Limited availability of fish meal and whey protein concentrate increases overall feed costs. Availability of increased number of supplemental amino acids including Lys, Met, Thr, Trp, Val, and Ile allows replacing expensive protein supplements to reduce feed costs. This study was to evaluate the effect of replacing fish meal and/or whey protein concentrate in nursery diets with 6 supplemental amino acids on growth performance and gut health of post-weaning pigs. Treatments were 1) FM-WPC: diet with fish meal (FM) and whey protein concentrate (WPC); 2) FM-AA: diet with FM and crystalline amino acids (L-Lys, L-Thr, L-Trp, DL-Met, L-Val, and L-Ile); 3) WPC-AA: diet with WPC and crystalline amino acid; and 4) AA: diet with crystalline amino acid.

**Results:**

Pigs in FM-AA, WPC-AA, and AA had greater (*P* < 0.05) ADG and gain:feed than pigs in FM-WPC during wk 1 (phase 1). Plasma insulin concentration of pigs in AA tended to be greater (*P* = 0.064) than that of FM-WPC at the end of wk 1(phase 1). Plasma concentrations of IgG in AA was lower (*P* < 0.05) compared with WPC-AA and FW, and FM-AA had lower (*P* < 0.05) IgG concentration than WPC-AA at the end of wk 1 (phase 1). Concentration of acetate in cecum digesta in FM-AA tended to be greater (*P* = 0.054) than that of FM-WPC and WPC-AA. Concentration of isovalerate in cecum digesta of pigs in FM-AA was greater (*P* < 0.05) than that of FW and WPC-AA.

**Conclusions:**

This study indicates that use of 6 supplemental amino acids can replace fish meal and/or whey protein concentrate without adverse effects on growth performance, immune status, and gut health of pigs at d 21 to 49 of age. Positive response with the use of 6 supplemental amino acids in growth during the first week of post-weaning may due to increased plasma insulin potentially improving uptake of nutrients for protein synthesis and energy utilization. The replacement of fish meal and/or whey protein concentrate with 6 supplemental amino acids could decrease the crude protein level in nursery diets, and potentially lead to substantial cost savings in expensive nursery diets.

## Background

Extremely high price of fish meal and whey protein concentrate increases overall cost of pork production. Because of advances in biotechnology, the use of supplemental amino acids in animal feeding are extended to Lys, Met, Thr, Trp, Val, Gln, and possibly Ile. Utilization of these essential amino acids as feed additives benefits swine industry by allowing nutritionists to use high levels of supplemental amino acids to replace high cost protein sources [1]; reduce crude protein levels in the diets [2–5]; formulate diets based on ideal protein concept [6–11]; reduce environmental excretion of nitrogen [3, 12, 13]; and reduce metabolic stress of pigs to detoxify excess ammonia [4, 14, 15].

Fish meal and whey protein concentrate are 2 of the most widely used animal protein sources in diets for nursery pigs. They are often included in these diets due to their high amino acid content and digestibility, which many believe eases the transition from sow’s milk to solid food for nursery pigs [16, 17]. However, the prices and availability for these ingredients have become extremely variable in recent years which increases overall cost of pork production and puts pressure on finding alternatives in case of limited availability.

Considering that the objective of a nursery diet is to provide a highly digestible and palatable diet to these young pigs in hopes of getting them to begin consuming feed sooner, perhaps there are opportunities to explore the use of lower crude protein diets supplemented with amino acids. The use of supplemental amino acids would offset or minimize the need to use some of expensive animal proteins, which could reduce the cost of these diets. Furthermore, extensive use of supplemental amino acids would allow to more precisely meet the pigs’s dietary requirements while reducing dietary crude protein. This change in formulation can positively impact pig health and the environment by reduction of environmental excretion of nitrogen and reduce metabolic stress of pigs to detoxify excess ammonia. Therefore, the objective of this study was to evaluate the effect of replacing fish meal and/or whey protein concentrate in nursery diets with 6 supplemental amino acids on growth performance, immune status, and gut health of post-weaning pigs.

## Methods

Experimental protocols were approved by the Institutional Animal Care and Use Committee at North Carolina State University (Raleigh, USA).

### Animals and experimental design

A total of 160 newly weaned pigs (6.49 ± 0.57 kg) at 21 d of age were used in a randomized complete block design with 4 dietary treatments, 8 replicates (4 barrow pens and 4 gilt pens) per treatment, and 5 pigs per pen. Treatments were 1) FM-WPC: diet with fish meal (**FM**) and whey protein concentrate (**WPC**); 2) FM-AA: diet with FM and supplemental amino acids (L-Lys, L-Thr, L-Trp, DL-Met, L-Val, and L-Ile); 3) WPC-AA: diet with WPC and supplemental amino acid; and 4) AA: diet with supplemental amino acid. All diets were formulated according to the nutrient requirements of NRC [18] (Tables 1 and 2). Pigs were fed the assigned experimental diets for a 4-wk period based on a 2-phase-feeding (phase 1: wk 1 post-weaning, Table 1; phase 2: wk 2 to 4 post-weaning, Table 2). During the 4-wk feeding period, all pigs were housed in a temperature-controlled nursery room (25 to 28°C). Pigs had access to diets and water *ad libitum* during the entire experimental period. Fresh feed was provided everyday. At 0800 h, any feed left from the previous day was removed and weighed. Feed intake and body weight were measured on d 7, 14, 21, and 28 post-weaning for determination of ADG, ADFI, and gain:feed.

**Table 1:** **Experimental diets for the study (phase 1)**

Items, %	FM-WPC ^1^	FM-AA ^2^	WPC-AA ^3^	AA ^4^
Corn grain	40.75	47.66	43.44	50.20
Soybean meal, without hulls	11.00	11.00	11.00	11.00
Whey powder	25.00	25.00	25.00	25.00
Plasma protein^5^	5.00	5.00	5.00	5.00
Whey protein concentrate	9.35	0.00	9.35	0.00
Fish meal, Menhaden	5.00	5.00	0.00	0.00
DL-Met	0.00	0.11	0.08	0.19
L-Thr	0.00	0.18	0.10	0.28
L-Val	0.00	0.15	0.11	0.26
L-Trp	0.00	0.05	0.02	0.08
L-Ile	0.00	0.15	0.10	0.26
L-Lys^6^	0.00	0.50	0.40	0.90
Aureomycin100	0.05	0.05	0.05	0.05
Zinc oxide	0.25	0.25	0.25	0.25
Vitamin-mineral premix^7^	0.50	0.50	0.50	0.50
Dical P	0.00	0.40	0.80	1.20
Ground limestone	0.70	0.80	0.90	1.00
Salt	0.40	0.40	0.40	0.40
Poultry fat	2.00	2.80	2.50	3.43
Total	100.00	100.00	100.00	100.00
Chemical composition:				
DM %	91.2	90.8	91.2	90.7
ME, kcal/kg	3411	3410	3409	3412
CP, %	22.56	20.76	20.17	18.45
SID^8^ Lys, %	1.34	1.34	1.34	1.34
SID Met, %	0.68	0.68	0.68	0.68
SID Trp, %	0.26	0.26	0.26	0.26
SID Thr, %	0.93	0.93	0.93	0.93
SID Val, %	0.99	0.99	0.99	0.99
SID Ile, %	0.78	0.78	0.78	0.78
Ca, %	0.91	0.92	0.90	0.91
Available P, %	0.56	0.56	0.56	0.55
Total P, %	0.70	0.72	0.70	0.71
Analyzed value:				
CP, DM basis, %	24.5	21.5	22.2	20.3

^1^FM-WPC: a control diet with fish meal and whey protein concentrate.

^2^FM-AA: a diet with fish meal and amino acids replacing whey protein concentrate.

^3^WPC-AA: a diet with whey protein concentrate and amino acids replacing fish meal.

^4^AA: a diet with amino acids replacing both fish meal and whey protein concentrate.

^5^APC-920 (APC Inc).

^6^Biolys (Evonik-Degussa).

^7^Vitamin-mineral premix provided the following per kilogram of complete phase 1 diet: 62.2 mg of manganese as manganese oxide, 100 mg of iron as iron sulfate, 138.4 mg of zinc as zinc sulfate, 12.7 mg of copper as copper oxide, 0.96 mg of iodide as ethylenediamine dihydroiodide, 0.3 mg of selenium as sodium selenite, 10,072 IU of vitamin A as vitamin A acetate, 1,100 IU of vitamin D3, 82.5 IU of vitamin E, 5.9 IU of vitamin K as menadione sodium bisulfite, 73.2 μg of vitamin B12, 18.3 mg of riboflavin, 58.5 mg of d-pantothenic acid as calcium pantothenate, 73.2 mg of niacin, and 6.4 g of choline as choline chloride.

^8^SID: Standardized ileal digestible.

**Table 2:** **Experimental diets for the study (phase 2)**

Items, %	FM-WPC ^1^	FM-AA ^2^	WPC-AA ^3^	AA ^4^
Corn grain	50.00	54.15	51.54	55.65
Soybean meal, without hulls	20.00	20.00	20.00	20.00
Whey powder	15.00	15.00	15.00	15.00
Plasma protein^5^	3.00	3.00	3.00	3.00
Whey protein concentrate	5.60	0.00	5.60	0.00
Fish meal, Menhaden	3.00	3.00	0.00	0.00
DL-Met	0.00	0.07	0.05	0.12
L-Thr	0.00	0.11	0.06	0.17
L-Val	0.00	0.09	0.07	0.16
L-Trp	0.00	0.04	0.02	0.05
L-Ile	0.00	0.09	0.06	0.15
L-Lys^6^	0.00	0.30	0.25	0.55
Aureomycin100	0.05	0.05	0.05	0.05
Vitamin-mineral premix^7^	0.50	0.50	0.50	0.50
Dical P	0.20	0.45	0.65	0.90
Ground limestone	0.90	0.95	1.05	1.10
Salt	0.30	0.30	0.30	0.30
Poultry fat	1.45	1.90	1.80	2.30
Total	100.00	100.00	100.00	100.00
Chemical composition:				
DM %	90.3	90.0	90.3	90.1
ME, kcal/kg	3,394	3,392	3,394	3,394
CP, %	22.0	21.0	20.7	19.6
SID^8^ Lys, %	1.19	1.19	1.19	1.19
SID Met, %	0.64	0.64	0.64	0.64
SID Trp, %	0.24	0.24	0.24	0.24
SID Thr, %	0.80	0.80	0.80	0.80
SID Val, %	0.91	0.91	0.91	0.91
SID Ile, %	0.76	0.76	0.76	0.76
Ca, %	0.82	0.82	0.82	0.82
Available P, %	0.41	0.41	0.41	0.41
Total P, %	0.61	0.62	0.61	0.62
Analyzed value:				
CP, DM basis, %	23.6	19.9	19.2	18.8

^1^FM-WPC: a control diet with fish meal and whey protein concentrate.

^2^FM-AA: a diet with fish meal and amino acids replacing whey protein concentrate.

^3^WPC-AA: a diet with whey protein concentrate and amino acids replacing fish meal.

^4^AA: a diet with amino acids replacing both fish meal and whey protein concentrate.

^5^APC-920 (APC Inc).

^6^Biolys (Evonik-Degussa).

^7^Vitamin-mineral premix provided the following per kilogram of complete phase 1 diet: 62.2 mg of manganese as manganese oxide, 100 mg of iron as iron sulfate, 138.4 mg of zinc as zinc oxide, 12.7 mg of copper as copper oxide, 0.96 mg of iodide as ethylenediamine dihydroiodide, 0.3 mg of selenium as sodium selenite, 10,072 IU of vitamin A as vitamin A acetate, 1,100 IU of vitamin D3, 82.5 IU of vitamin E, 5.9 IU of vitamin K as menadione sodium bisulfite, 73.2 μg of vitamin B12, 18.3 mg of riboflavin, 58.5 mg of d-pantothenic acid as calcium pantothenate, 73.2 mg of niacin, and 6.4 g of choline as choline chloride.

^8^SID: Standardized ileal digestible.

### Experimental diets

The phase 1 and 2 diets are shown in Tables 1 and 2. All diets contained soybean meal and plasma protein, and the inclusions of these ingredients were held constant across all diets in each growth phase. Soybean meal contents were 11.0 and 20.0% for phase 1 and 2, and plasma protein contents for phase 1 and 2 were 5.0 and 3.0%, respectively. Crude protein content was various among treatment diets. Fish meal (5.0 and 3.0% for phase 1 and 2, respectively) and whey protein concentrate (9.35 and 5.60% for phase 1 and 2, respectively) were used depending on treatment diets. Amino acid contents were measured in triplicate for soybean meal, fish meal, whey protein concentrate, and plasma protein from the same batch to be used for this study (12 samples). These data were used to formulate the diets. All diets were formulated to contain the same standardized ileal digestible levels of Lys, Thr, Trp, Met, Val, and Ile for each respective phase, and all nutrients met or exceeded the NRC [18] requirements. After the diet formulation, amino acid contents in each diet were measured in triplicate to check if diets were formulated to meet the target levels (12 samples). The results showed that all diets had the same content (standard ileal digestible amount basis) of Lys, Met, Thr, Trp, Val, and Ile (Table 3).

**Table 3:** **Growth performance of pigs fed experimental diets**

Items	FM-WPC ^1^	FM-AA ^2^	WPC-AA ^3^	AA ^4^	SEM	***P*** value
n	8	8	8	8		
Age, d	22.1	22.7	23.3	22.5	0.6	NS^5^
Body weight, kg						
Initial	6.50	6.53	6.54	6.38	0.57	NS
Wk 1	7.30	7.74	7.96	7.65	0.58	NS
Wk 2	8.70	9.46	9.52	9.20	0.66	NS
Wk 3	11.07	11.83	11.87	11.50	0.66	NS
Wk 4	15.11	15.85	15.69	16.04	0.87	NS
ADG, g						
Wk 1	114.3^b^	172.6^a^	202.7^a^	182.0^a^	15.2	0.031
Wk 2	200.1	246.5	223.3	221.4	19.9	NS
Wk 3	338.8	338.3	336.3	328.1	26.1	NS
Wk 4	576.1	574.6	545.4	648.0	44.1	NS
Wk 1 to 4	307.3	333.0	326.9	344.9	14.1	NS
ADFI, g						
Wk 1	198.5	235.0	240.4	237.2	15.4	NS
Wk 2	303.0	318.5	326.4	325.0	16.7	NS
Wk 3	539.9	538.7	566.1	524.4	22.9	NS
Wk 4	1,106.1	1,106.6	1,109.1	1,168.2	33.5	NS
Wk 1 to 4	525.6	539.5	556.0	563.7	11.5	NS
Gain:feed						
Wk 1	0.562^b^	0.730^a^	0.836^a^	0.774^a^	0.045	0.008
Wk 2	0.664	0.766	0.681	0.684	0.044	NS
Wk 3	0.634	0.625	0.595	0.625	0.044	NS
Wk 4	0.566	0.551	0.514	0.565	0.055	NS
Wk 1 to 4	0.592	0.618	0.590	0.614	0.026	NS

^1^FM-WPC: a control diet with fish meal and whey protein concentrate.

^2^FM-AA: a diet with fish meal and amino acids replacing whey protein concentrate.

^3^WPC-AA: a diet with whey protein concentrate and amino acids replacing fish meal.

^4^AA: a diet with amino acids replacing both fish meal and whey protein concentrate.

^5^NS: not significant (*P* > 0.10).

^a-b^Means lacking a common superscript are differ within a row (*P* < 0.05).

### Blood cell counts

At the end of each growth phase, blood sample from one pig per pen (same pig was used in both phases) was collected from the jugular vein using 10-mL heparin-coated blood tube and disposable 22-gauge × 0.025 mm Vacutainer needle (BD, Franklin Lakes, NJ). Hematocrit, hemoglobin, mean corpuscular hemoglobin, mean corpuscular hemoglobin concentration, mean corpuscular volume, mean platelet volume (**MPV**), platelet number, red blood cell count, red blood cell distribution width, white blood cell (**WBC**) count, neutrophils, lymphocytes, monocytes, eosinophils, and basophils in whole blood samples were used for hematological evaluation using an automated hematological analyzer (Cell-Dyn 6300, Abbott Diagnostics, Abbott Park, IL). Plasma samples were then collected by centrifuging (5810 R, Eppendorf AG, Hamburg, Germany) at 3,000 g, 15 min, 4°C, allocated into 1.5 mL microcentrifuge tubes, and stored at -80°C for analysis. Plasma from blood was later analyzed for the contents of immunoglobulin G (**IgG**), immunoglobulin A (**IgA**), tumor necrosis factor α (**TNF-α**) at the end of phase 1, and insulin and growth hormone at the end of phase 1 and 2.

### Immune responses

At the end of phase 1, plasma samples were used to measure IgG, IgA, and TNF-α. Total concentrations of IgG and IgA in plasma of pigs were measured according to the method described by Chaytor et al. [19] using ELISA kits (Bethyl, Montgomery, TX). Firstly, goat anti-pig IgG or IgA were used as capture antibodies to coat wells. Then plasma samples were diluted to 1:100,000 for the measurement. Horseradish peroxidase goat anti-pig IgG or IgA was used as the detection. The plate was read at 450 nm by an ELISA plate reader (Synergy HT, BioTek Instruments, Winooski, VT) and software (KC4 Data Analysis Software, BioTek Tnstrument). Sample concentration was quantified against the standard curve. Detective limits were 7.8 ng/mL for IgG, and 15.6 ng/mL for IgA, respectively.

Determination of TNF-α concentrations in plasma was completed according to the method described by Chaytor et al. [19] using ELISA kits (Pierce Biotechnology, Rockford, IL). Briefly, 50 μL of standard plus dilute or 100 μL of sample was added to microplate wells coated with capture antibody in conjunction with biotinylated antibody reagent. Horseradish peroxidase, TMB substrate, and a stop solution of 2 mol/L H_2_SO_4_ were used for the detection. Absorbance was read at 450 nm by an ELISA plate reader (Synergy HT, BioTek Instruments, Winooski, VT) and software (KC4 Data Analysis Software, BioTek Tnstrument). Detection limit for TNF-α was 5 pg/mL.

### Growth hormone

Plasma growth hormone concentration was determined according to the method described by Atinmo et al. [20] using the porcine/canine growth hormone radioimmunoassay kit (Millipore, Charles, MO). Briefly, label total count tubes (tube 1-2), non-specific binding (**NSB**) tubes (tube 3-4), reference tubes (tube 5-6), and sample tubes (from tube 7). Assay buffer was first added into the NSB tubes, reference tubes, and sample tubes followed adding standards, or quality controls, or plasma samples. Then ^125^I-porcine/canine growth hormone was added to all tubes. Guinea pig porcine/canine antiserum was added to all tubes except total count tubes and NSB tubes. After incubation overnight at room temperature, cold (4°C) precipitating reagent was added to all tubes (except total count tubes). After incubation and centrifugal separation, immediately decant the supernate of all tubes except total count tubes, and drain tubes for at least 15 to 60 s. An automatic gamma counter (WIZARD, PerkinElmer, Waltham, MA) was used to count all tubes and calculate the concentration of porcine/canine growth hormone in plasma samples. The limit of sensitivity for the porcine/canine growth hormone assay was 1 ng/mL.

### Insulin

Plasma insulin was determined using a commercial ELISA kit (Mercodia AB, Uppsala, Sweden), which allowed for direct measurement of plasma using an ELISA plate reader (Synergy HT, BioTek Instruments, Winooski, VT) and software (KC4 Data Analysis Software, BioTek Tnstrument). All samples were tested in duplicate. Detection limit for insulin was 0.007 ng/mL.

### Gut health

At the end of phase 1, one pig from each pen (from 4 barrow pens and 4 gilt pens per treatment, n = 8) were selected and euthanized at 10 am to collect intestinal tissues and digesta samples. After killing, gastrointestinal tract with digesta were removed. The representative digesta from cecum and colon were gently squeezed into a tube and then stored at -80°C until analysis for volatile fatty acids (**VFA**) concentrations [21, 22]. Intestinal tissue from middle section of jejunum was isolated, flushed with 0.9% salt solution, fixed in 10% formaldehyde-phosphate buffer, and stored at 4°C for microscopic assessment of mucosal morphology. Briefly, the fixed intestinal segments were embedded in paraffin wax. Cross sections of 6 μm thickness of each intestinal segments were obtained, and stained with hematoxylin and eosin. Villus height was determined at 40× magnification as described by Shen et al. [22]. Villous height from the tip to the crypt-villus junction on 10 well-oriented villi were measured in triplicate for each intestinal segment of each pig [22, 23].

### Statistical analysis

All the data were analyzed by the GLM procedure (SAS Inst., Inc., Cary, NC) following a randomized complete block design with dietary treatment as the main effect and sex as a block. The pen was the experimental unit. Separation of means was done using the PDIFF option of SAS. Treatment effects were considered statistically significant when probability values were less than 0.05, and probability less than 0.1 and equal or greater than 0.05 was considered as a trend.

## Results

As there was no effect (*P* > 0.10) of sex in all measurement, sex effect was excluded in the result. Age and initial body weight of pigs did not differ among treatments (Table 3). Body weights of pigs did not differ among treatments at the end of wk 1, 2, 3, and 4. Average daily gain of pigs did not differ among treatments during wk 2, 3, 4, and the entire 4-wk period. However, pigs in FM-AA, WPC-AA, and AA had greater (*P* < 0.05) ADG than pigs in FM-WPC during wk 1 (phase 1). Average daily feed intake did not differ among treatments during wk 1, 2, 3, 4, and the entire 4-wk period. Gain:feed did not differ among treatments during wk 2, 3, 4, and the entire 4-wk period. However, pigs in FM-AA, WPC-AA, and AA had greater (*P* < 0.05) gain:feed than pigs in FM-WPC during wk 1 (Table 3).

Plasma concentrations of growth hormone did not differ among treatments when measured at the end of wk 1 and 4 (Table 4). Plasma insulin concentration of pigs in AA tended to be greater (*P* = 0.064) than that of FM-WPC at the end of wk 1. However, plasma insulin concentration of pigs did not differ among treatments at the end of wk 4. Plasma concentrations of IgM were not different among treatments at the end of wk 1. Plasma concentrations of IgG in AA was similar to FM-AA, but was lower (*P* < 0.05) compared with WPC-AA and FM-WPC, and FM-AA had lower (*P* < 0.05) IgG concentration than WPC-AA at the end of wk 1. Plasma concentration of TNF-α did not differ among treatments at the end of wk 1 (Table 4). There was no treatment × sex interaction for all plasma measurements.

**Table 4:** **Plasma mearurements of pigs fed experimental diets**

Items	FM-WPC ^1^	FM-AA ^2^	WPC-AA ^3^	AA ^4^	SEM	***P*** value
n	8	8	8	8		
Phase 1, d 7						
Growth hormone, ng/mL	9.205	5.277	7.232	8.225	2.170	NS^5^
Insulin, μg/L	0.026^A^	0.034^AB^	0.028^AB^	0.058^B^	0.012	0.064
Immunoglobulin G, ug/mL	0.325^ab^	0.290^bc^	0.362^a^	0.275^c^	0.028	0.024
Immunoglobulin M, ug/mL	0.058	0.060	0.066	0.060	0.006	NS
TNF-α, pg/mL	47.0	47.3	35.0	47.3	8.9	NS
Phase 2, d 28						
Growth hormone, ng/mL	4.163	4.140	6.251	7.298	1.686	NS
Insulin, μg/L	0.026	0.049	0.034	0.024	0.011	NS

^1^FM-WPC: a control diet with fish meal and whey protein concentrate.

^2^FM-AA: a diet with fish meal and amino acids replacing whey protein concentrate.

^3^WPC-AA: a diet with whey protein concentrate and amino acids replacing fish meal.

^4^AA: a diet with amino acids replacing both fish meal and whey protein concentrate.

^5^NS: not significant (*P* > 0.10).

^a-b^Means lacking a common superscript are differ within a row (*P* < 0.05).

^A-B^Means lacking a common superscript tend to differ within a row (0.05 ≤ *P* < 0.1).

Numbers of blood cells and hematological characteristics were not different among treatments at the end of wk 1 and wk 4 (Table 5). Height of villi in jejunum of pigs did not differ among treatments at the end of wk 4 (Table 6). Concentration and percentage of propionate, isobutyrate, butyrate, and valerate in digesta did not differ among treatments at the end of wk 4 (Table 6). However, acetate concentration in cecum digesta of pigs in FM-AA tended to be greater (*P* = 0.054) than that of FM-WPC and WPC-AA (Table 6). Concentration of isovalerate in cecum digesta of pigs in FM-AA was greater (*P* < 0.05) than that of FM-WPC and WPC-AA. Percentage of isovalerate in total volatile fatty acids in colon digesta of pigs in FM-AA tended to be greater (*P* = 0.057) than that of WPC-AA (Table 6).

**Table 5:** **Hematological evaluation of pigs fed experimental diets**

Items	FM-WPC ^1^	FM-AA ^2^	WPC-AA ^3^	AA ^4^	SEM	***P*** value
n	8	8	8	8		
Phase 1, d 7						
White blood cell, 10^3^ cell/μL	13.09	12.78	13.81	13.83	1.16	NS^5^
Red blood cell, 10^6^ cell/μL	6.18	6.14	5.93	6.23	0.28	NS
Hemoglobin, g/dL	10.60	10.64	9.99	10.76	0.64	NS
Hematocrit, %	33.60	34.23	32.56	34.61	1.77	NS
Mean corpuscular volume, fL	54.25	56.11	55.11	55.54	1.79	NS
Mean corpuscular hemoglobin, pg/cell	17.09	17.46	16.93	17.21	0.73	NS
Mean corpuscular hemoglobin concentration, %	31.40	31.03	30.64	30.95	0.40	NS
Red blood cell distribution width, μm	18.13	19.71	20.73	20.80	1.78	NS
Platelet count, /μL	407.9	393.8	346.6	439.6	57.0	NS
Mean platelet volume, fL	10.24	9.84	10.28	10.61	0.44	NS
Neutrophil, 10^3^ cell/μL	4.86	5.78	6.05	6.16	0.65	NS
Lymphocyte, 10^3^ cell/μL	7.13	5.72	6.41	6.50	0.65	NS
Monocyte, 10^3^ cell/μL	0.59^A^	0.62^A^	0.91^B^	0.56^A^	0.09	0.057
Eosinophil, 10^3^ cell/μL	0.23	0.33	0.41	0.29	0.08	NS
Basophil, 10^3^ cell/μL	0.05	0.08	0.06	0.06	0.04	NS
Phase 2, d 28						
White blood cell, 10^3^ cell/μL	17.99	14.51	18.74	18.46	1.863	NS
Red blood cell, 10^6^ cell/μL	6.01^ab^	5.93^a^	5.96^a^	6.15^b^	0.09	0.005
Hemoglobin, g/dL	10.05^ab^	10.27^a^	9.88^b^	10.30^a^	0.08	0.002
Hematocrit, %	31.65	32.11	30.54	32.59	0.73	NS
Mean corpuscular volume, fL	52.96	54.53	51.38	53.04	1.45	NS
Mean corpuscular hemoglobin, pg/cell	16.84	17.43	16.63	16.79	0.50	NS
Mean corpuscular hemoglobin concentration, %	31.75	31.96	32.30	31.63	0.23	NS
Red blood cell distribution width, μm	18.50	19.27	19.90	19.31	1.11	NS
Platelet count, /μL	467.0	453.1	357.6	506.9	60.2	NS
Mean platelet volume, fL	9.75	9.49	9.56	9.81	0.46	NS
Neutrophil, 10^3^ cell/μL	5.79^A^	5.33^A^	8.02^B^	5.95^A^	1.52	0.082
Lymphocyte, 10^3^ cell/μL	10.15	7.33	8.61	10.42	0.91	NS
Monocyte, 10^3^ cell/μL	1.34	1.02	1.02	1.16	0.18	NS
Eosinophil, 10^3^ cell/μL	0.43	0.37	0.42	0.38	0.06	NS
Basophil, 10^3^ cell/μL	0.11	0.10	0.13	0.12	0.02	NS

^1^FM-WPC: a control diet with fish meal and whey protein concentrate.

^2^FM-AA: a diet with fish meal and amino acids replacing whey protein concentrate.

^3^WPC-AA: a diet with whey protein concentrate and amino acids replacing fish meal.

^4^AA: a diet with amino acids replacing both fish meal and whey protein concentrate.

^5^NS: not significant (*P* > 0.10).

^A-B^Means lacking a common superscript tend to differ within a row (0.05 ≤ *P* < 0.1).

^a-d^Means lacking a common superscript are differ within a row (*P* < 0.05).

**Table 6:** **Villus height and concentrations of volatile fatty acids in digesta of pigs fed experimental diets (d 28)**

Items	FM-WPC ^1^	FM-AA ^2^	WPC-AA ^3^	AA ^4^	SEM	***P*** value
n	8	8	8	8		
Villus height, μm	376.8	477.8	505.4	472.8	42.2	0.208
Cecum digesta						
Acetate, mmol/L	66.04^A^	94.63^B^	65.24^A^	90.86^AB^	10.74	0.054
Propionate, mmol/L	40.94	50.15	39.25	47.51	8.37	NS^5^
Isobutyrate, mmol/L	0.54	0.98	0.40	0.83	0.22	NS
Butyrate, mmol/L	18.16	22.30	14.41	25.63	4.45	NS
Isovalerate, mmol/L	0.41^a^	0.82^b^	0.30^a^	0.58^ab^	0.12	0.032
Valerate, mmol/L	2.99	2.70	2.22	4.38	0.67	NS
Total, mmol/L	129.06	171.57	121.79	169.78	21.78	NS
Acetate, % of total	53.21	56.15	54.45	52.55	3.40	NS
Propionate, % of total	29.05	28.67	32.27	28.81	3.51	NS
Isobutyrate, % of total	0.31	0.61	0.27	0.45	0.13	NS
Butyrate, % of total	13.95	12.38	11.03	15.35	1.81	NS
Isovalerate, % of total	0.24	0.55	0.22	0.32	0.10	NS
Valerate, % of total	3.24	1.65	1.77	2.53	0.73	NS
Colon digesta						
Acetate, mmol/L	92.90	110.52	93.78	117.08	16.81	NS
Propionate, mmol/L	50.58	54.17	52.10	51.78	10.32	NS
Isobutyrate, mmol/L	1.76	1.18	1.69	1.81	0.36	NS
Butyrate, mmol/L	22.67	25.16	20.98	28.60	5.43	NS
Isovalerate, mmol/L	1.99	1.93	1.11	1.44	0.44	NS
Valerate, mmol/L	5.34	3.80	3.70	4.00	1.20	NS
Total, mmol/L	175.22	196.75	173.34	204.68	31.82	NS
Acetate, % of total	54.64	57.20	53.56	57.23	2.51	NS
Propionate, % of total	26.45	26.82	31.14	25.35	2.88	NS
Isobutyrate, % of total	0.77	0.64	0.94	0.88	0.17	NS
Butyrate, % of total	13.36	12.30	11.71	13.90	1.68	NS
Isovalerate, % of total	0.85^AB^	1.05^B^	0.59^A^	0.69^AB^	0.18	0.057
Valerate, % of total	3.95	2.00	2.07	1.95	0.73	NS

^1^FM-WPC: a control diet with fish meal and whey protein concentrate.

^2^FM-AA: a diet with fish meal and amino acids replacing whey protein concentrate.

^3^WPC-AA: a diet with whey protein concentrate and amino acids replacing fish meal.

^4^AA: a diet with amino acids replacing both fish meal and whey protein concentrate.

^5^NS: not significant (*P* > 0.10).

^a-b^Means lacking a common superscript are differ within a row (*P* < 0.05).

^A-B^Means lacking a common superscript tend to differ within a row (0.05 ≤ *P* < 0.1).

## Discussion

The reduction in growth rate during the period immediately after weaning is a major problem in nursery pig nutrition and management. It was hypothesized that supplementing highly digestible crystalline amino acids to nursery diets could support growth performance, minimize the need for the use some of expensive animal proteins such as fish meal and/or WPC, and reduce the crude protein level in diets to reduce the cost [4]. Our data demonstrate that supplementing amino acids to replace fish meal and/or WPC in nursery diet increased ADG and gain:feed than pigs in FM-WPC during wk 1 (phase 1). This is in agreement with the result of previous study [12]. In this study, we did not observe changes in voluntary feed intake, indicating that the amount of energy and standardized amino acids provided for utilization did not differ among treatments, therefore feed intake was not the main reason for the increased ADG and feed:gain in phase 1 but rather it could be contributed from enhanced amino acid availability and reduced energy costs for digestion of crude protein. True digestibilities of supplemental amino acids were often found to be 100% digestible [24]. The average apparent ileal digestibilities of fish meal was reported to be a approximately 73.6% [6]. In this study, although all diets were formulated to contain the same standardized ileal digestible levels of Lys, Thr, Trp, Met, Val, and Ile, the different digestibilities of amino acid from supplemented amino acid source or fish meal source could result in the difference of amino acid digestion and absorption between FM-WPC and amino acid supplemented treatments in phase 1. In addition, plasma insulin concentration of pigs in AA in this study tended to be greater than that of FM-WPC at the end of wk 1 (phase 1). Amino acids and insulin are critical regulators of muscle protein synthesis in neonatal pigs [25]. Thus, the improved growth performance was possibly induced by enhanced insulin sensitivity due to increased plasma free amino acid contents.

When supplementing amino acid to replace fish meal and/or WPC in phase 2 nursery diets, although there was numerically increase in ADG and gain:feed in amino acid supplemented treatments compared with FM-WPC, statistically there was no difference for growth performance among treatments. This is in agreement with previous studies [2, 26, 27]. Nyachoti et al. [4] showed that daily feed intake, ADG, and G:F of post-weaning pigs were not affected by reducing CP level from 23% to 21%, however, they also reported that piglet performance may suffer when dietary CP level was reduced by 4 or more percentage units from 23%. Results in this study indicate that replacing fish meal and/or WPC with crystalline amino acid may improve growth performance of pigs immedically after weaning. When CP level was reduced to 20% by supplementing amino acid during the nursery phase, the growth performance was not affected.

This study also examed the plasma concentrations of growth hormone which did not differ among treatments when measured at the end of wk 1 and 4. Variations among values of growth hormones were rather large which may have contributed to no significant differences among means. The result indicates that supplemented amino acid to replace fish meal and/or WPC in nursery diets did not affect growth hormone changes in plasma of pigs.

In this study, supplemental amino acid to fish meal and/or WPC improve ADG and gain:feed in phase 1 diet without adversely affecting immune status of pigs in phase1, as showing by plasma concentrations of IgM and TNF-α which were not different among treatments at the end of wk 1. In addition, plasma concentrations of IgG was lower in AA compared with FM-WPC at the end of wk 1. The reduced immune challenge could be another reasons for the positive effect of dietary supplementation of the amino acid on pig’s growth performance in wk 1.

To obtain further knowledge about how supplemental amino acids affect the post-weaning pigs, the blood cell counts and plasma composition were measured during 4-wk study. Results indicate that supplemented amino acids did not affect numbers of blood cells and hematological characteristics among treatments at the end of wk 1 and wk 4. Numbers of white blood cells and red blood cells, neutrophils, lymphocytes, monocytes, eosinophils, and basophils in pigs are shown as normal compared with reference values suggested by Friendship et al. [28], Dubreuil and Lapierre [29], and Klem et al. [30].

Weaning is one of the most critical developmental stages of the digestive tract of pigs when food is changed from liquid milk to a solid diet. The process of weaning could be divided into the acute phase and adaptive phase. During the acute phase, there is a significant reduction in villus height in the small intestine, causing severe damages to intestinal health and function [31, 32]. Nyachoti et al. [4] reported that supplemented amino acid in nursery diet did not affect gut morphology of post-weaning pigs. In this study, we also did not observe an improvement in the height of villi in jejunum of pigs in amino acid supplemented treatments compared with FM-WPC at the end of wk 4.

In this study, VFA concentrations were used as an index of the changes in microbial fermentation in colon and cecal digest samples. The VFA, also known as short-chain fatty acids, are produced in pigs by microbial fermentation of undigested nutrients such as protein and carbohydrates in the gastrointestinal tract [4, 33]. The degree of fermentation depends on the presence of nitrogen, minerals, vitamins, and primarily upon the source of dietary fiber which are essential for the microbial populations residing in the hindgut [33]. Diets for early-weaned pigs usually contain high levels of protein, which may enhance proliferation of pathogenic bacteria in the gastrointestinal tract [4]. It was shown that ammonia N in ileal digesta was reduced linearly as dietary CP was decreased, indicating that low CP diets help maintain enteric health in pigs by lowering toxic microbial metabolites such as ammonia [4]. Therefore, in the current study, it was anticipated that feeding post-weaning pigs with supplemented amino acid diets to reduce CP level would minimize microbial proliferation in the hindgut and also limit their byproducts such as VFA, thus giving pigs an opportunity to better utilize dietary nutrients for growth. However, results in this study showed that concentration of acetate in cecum digesta of pigs in FM-AA was the same to WPC-AA and AA, but tended to be greater than that of FM-WPC. Concentration of isovalerate in cecum digesta of pigs in FM-AA was the same to AA, but was greater than that of FM-WPC and WPC-AA, indicating increased fermentation of hind gut in FM-AA group. This was opposite to our previous hypothesis. In this study, we did not observe changes in voluntary feed intake, indicating that the amount of energy and std AA were not different among treatment. However, it is important to note that the levels of dietary fiber in the current experimental diets changed with differences in CP content. Treatment FM-AA in phase 1 and 2 contained higher corn content than FM-WPC and WF, which means dietary fiber content could be higher than FM-WPC and WF. Because feed intake was not different among treatment, and dietary fiber affects microbial activities in the gut [4], especially in the cecum and colon, it could speculated that the higher fiber content supplied in FM-WPC could be the reason to cause increased fermentation in FM-AA treatment.

The principal VFA produced in either the rumen or large intestine are acetate, propionate, and butyrate, which are produced in a ratio varying from approximately 75:15:10 to 40:40:20 [33–35]. Franklin et al. [36] showed a ratio of 71 acetate: 28 propionate: 10 butyrate in cecum digesta of pigs. Grieshop et al. [33] reproted a ratio of 60 acetate: 20 propionate: 15 butyrate in colon digesta of pigs. These VFA compositions were comparable to our current measurements with an average ratio of 54 acetate: 30 propionate: 13 butyrate. These results demonstrate that supplementing amino acid to replace the use of fish meal and/or whey protein concentrate did not adversely affect gut morphology and health of pigs.

## Conclusions

This study demonstrates that supplemental L-Lys, L-Thr, L-Trp, DL-Met, L-Val, and L-Ile successfully replaced the use of fish meal and/or whey protein concentrate without adverse effects on growth performance, immune status, and gut health of pigs at d 21 to 49 of age. Positive response with supplemental amino acid in growth during the first week of post-weaning may be partially due to increased plasma insulin concentration potentially improving uptake of nutrients for protein synthesis and energy utilization. The replacement of fish meal and/or whey protein concentrate with supplemental amino acids could decrease the crude protein level in nursery diets, and potentially lead to substantial cost savings in expensive nursery diets.

## References

[1] Junghans P, BeyeR M, Derno M, Petzke KJ, Küchenmeister U, Hennig U, Jentsch W, Schwerin M (2007). Studies on persisting effects of soy-based compared with amino acid-supplemented casein-based diet on protein metabolism and oxidative stress in juvenile pigs. Arch Anim Nutr.

[2] Hansen JA, Knabe DA, Burgoon KG (1993). Amino acid supplementation of low-protein sorghum-soybean meal diets for 5- to 20-kilogram swine. J Anim Sci.

[3] Shriver JA, Carter SD, Sutton AL, Richert BT, Senne BW, Pettey LA (2003). Effects of adding fiber sources to reduced-crude protein, amino acidsupplemented diets on nitrogen excretion, growth performance, and carcass traits of finishing pigs. J Anim Sci.

[4] Nyachoti CM, Omogbenigun FO, Rademacher M, Blank G (2006). Performance responses and indicators of gastrointestinal health in early weaned pigs fed low-protein amino acid-supplemented diets. J Anim Sci.

[5] Eklund M, Mosenthin R, Piepho HP, Rademacher M (2008). Effect of dietary crude protein level on basal ileal endogenous losses and standardized ileal digestibilities of crude protein and amino acids in newly weaned pigs. J Anim Physol Anim Nutr.

[6] Sauer WC, Ozimek L (1986). Digestibility of amino acids in swine: Results and their practical applications. A review. Livest Prod Sci.

[7] Baker DH, Hahn JD, Chung TK, Hollis GR (1993). Nutrition and growth: The concept and application of an ideal protein for swine growth. Growth of the Pig.

[8] Mosenthina R, Sauerb WC, Blankc R, Huismand J, Fan MZ (2000). The concept of digestible amino acids in diet formulation for pigs. Livest Prod Sci.

[9] Kim SW, Baker DH, Easter RA (2001). Dynamic ideal protein and limiting amino acids for lactating sows: Impact of amino acid mobilization. J Anim Sci.

[10] Kim SW, Hurley WL, Wu G, Ji F (2009). Ideal amino acid balance for sows during gestation and lactation. J Anim Sci.

[11] Eklunda M, Mosenthina R, Piephob HP, Rademacher M (2010). Estimates of dietary threshold levels for crude protein and amino acids to obtain plateau values of apparent ileal crude protein and amino acid digestibilities in newly weaned pigs. Arch Anim Nutr.

[12] Jin CF, Kim IH, Han K, Bae SH (1998). Effects of supplemental synthetic amino acids to the low protein diets on the performance of growing pigs. Asian-Aus J Anim Sci.

[13] Dai ZL, Zhang J, Wu GY, Zhu WY (2010). Utilization of amino acids by bacteria from the pig small intestine. Amino Acids.

[14] Figueroa JL, Lewis AJ, Miller PS, Fischer RL, Gomez RS, Diedrichsen RM (2002). Nitrogen metabolism and growth performance of gilts fed standard corn-soybean meal diets or low-crude protein, amino acid supplemented diets. J Anim Sci.

[15] Columbus D, Cant JP, de Lange CFM (2010). Estimating fermentative amino acid losses in the upper gut of pigs. Livest Sci.

[16] Stoner GR, Allee GL, Nelssen JL, Johnston ME, Goodband RD (1990). Effect of select menhaden fish meal in starter diets for pigs. J Anim Sci.

[17] Grinstead GS, Goodband RD, Dritz SS, Tokach MD, Nelssen JL, Woodworth JC, Molitor M (2000). Effects of a whey protein product and spray-dried animal plasma on growth performance of weanling pigs. J Anim Sci.

[18] NRC (1998). Nutrient Requirements of Swine.

[19] Chaytor AC, See MT, Hansen JA, de Souza ALP, Middleton TF, Kim SW (2011). Effects of chronic exposure of diets with low levels of aflatoxin and deoxynivalenol on growth and immune status of pigs. J Anim Sci.

[20] Atinmo T, Baldijão C, Pond WG, Barnes RH (1976). Decreased dietary protein or energy intake and plasma growth hormone levels of the pregnant pig, its fetuses and developing progeny. J Nutr.

[21] Mathew AG, Chattin SE, Robbins CM, Golden DA (1998). Effects of a direct-fed yeast culture on enteric microbial populations, fermentation acids, and performance of weaning pigs. J Anim Sci.

[22] Shen YB, Piao XS, Kim SW, Wang L, Liu P, Yoon I, Zhen YG (2009). Effects of yeast culture supplementation on growth performance, intestinal health, and immune response of nursery pigs. J Anim Sci.

[23] Touchette KJ, Carroll JA, Allee GL, Matteri RL, Dyer CJ, Beausang LA, Zannelli ME (2002). Effect of spraydried plasma and lipopolysaccharide exposure on weaned pig diets.

[24] Chung TK, Baker DH (1992). Apparent and true amino acid digestibility of a crystalline amino acid mixture and of casein: comparison of values obtained with ileal-cannulated pigs and cecectomized cockerels. J Anim Sci.

[25] Wu GY (2009). Amino acids: metabolism, functions, and nutrition. Amino Acids.

[26] Southern LL, Baker DH (1982). Performance and concentration of amino acids in plasma and urine of young pigs fed diets with excesses of either arginine or lysine. J Anim Sci.

[27] Le Bellego L, Noblet J (2002). Performance and utilization of dietary energy and amino acids in piglets fed low protein diets. Livest Prod Sci.

[28] Friendship RM, Lumsden JH, McMillan I, Wilson MR (1984). Hematology and biochemistry reference values for Ontario swine. Can J Comp Med.

[29] Dubreuil P, Lapierre H (1997). Biochemistry reference values for Quebec lactating dairy cows, nursing sows, growing pigs and calves. Can J Vet Res.

[30] Klem TB, Bleken E, Morberg H, Thoresen SI, Framstad T (2009). Hematologic and biochemical reference intervals for Norwegian crossbreed grower pigs. Vet Clin Patho.

[31] Pluske JR, Williams IH, Aherne FX, Varley MA (1996). Nutrition of the neonatal pig. The Neonatal Pig: Development and Survival.

[32] Hou YQ, Wang L, Ding BY, Liu YL, Zhu HL, Liu J, Li YT, Wu X, Yin YL, Wu GY (2010). Dietary a-ketoglutarate supplementation ameliorates intestinal injury in lipopolysaccharide-challenged piglets. Amino Acids.

[33] Grieshop CM, Reese DE, Fahey GC, Lewis AJ, Southern LL (2000). Nonstarch polysaccharides and oligosaccharides in swine nutrition. Swine Nutrition.

[34] Rao SO (2007). Effects of dietary supplementation of Lactobacillus based probiotics on growth and gut environment of nursery pigs.

[35] Vasquez CM (2008). Effects of yeast culture supplementation to gestation and lactation diets on performance of sows and their offspring.

[36] Franklin MA, Mathew AG, Vickers JR, Clift RA (2002). Characterization of microbial populations and volatile fatty acid compositions in the jejunum, ileum, and cecum of pigs weaned at 17 vs. 24 days of age. J Anim Sci.

